# Relationship between Echocardiographic and Magnetic Resonance-Derived Measurements of the Thoracic Aorta in Turner Syndrome Patients

**DOI:** 10.1155/2019/9258726

**Published:** 2019-08-20

**Authors:** Rūta Krikščiūnienė, Inesa Navickaitė, Eglė Ereminienė, Saulius Lukoševičius, Birutė Žilaitienė, Rasa Verkauskienė

**Affiliations:** ^1^Lithuanian University of Health Sciences, Department of Endocrinology, Medical Academy, Kaunas, Lithuania; ^2^Lithuanian University of Health Sciences, Kaunas, Lithuania; ^3^Lithuanian University of Health Sciences, Department of Cardiology, Medical Academy, Kaunas, Lithuania; ^4^Lithuanian University of Health Sciences, Department of Radiology, Medical Academy, Kaunas, Lithuania

## Abstract

**Introduction:**

Turner syndrome (TS) is assigned to the rare diseases group. Morbidity and mortality of TS patients are high, particularly due to the cardiovascular disorders, so monitoring for cardiovascular complications must be ensured. The data demonstrate a strong correlation between 2-dimensional echocardiographic (2Decho) evaluation and magnetic resonance imaging (MRI); still, according to recent guidelines, MRI remains a gold standard. In this study, we aimed to compare aortic dimensions on MRI and 2Decho in TS patients.

**Methods:**

50 TS patients (≥18 years) were enrolled into the cross-sectional study. 2Decho and MRI were performed. The measurements of the aorta were assessed in five standard positions on 2Decho and in 9 standard positions on MRI; ASI (aortic size index) of the ascending aorta was calculated since reduced adult height is observed in TS patients.

**Results:**

ASI on echocardiography strongly correlated with ASI on MRI in all positions of the ascending aorta, but significantly larger medians of ASI were found on 2Decho in all positions of the ascending aorta and arch when compared with MRI measurements. Still, the prevalence of aortic sinus dilation was significantly and more frequently (52% vs. 38%, *p* < 0.001) observed on MRI when compared with 2Decho.

**Conclusion:**

The relation of aortic size was significant in all positions when comparing the MRI and 2Decho methods; still, the dilatation of the sinus of aorta was more frequently found on MRI compared with echocardiography.

## 1. Background

Turner syndrome (TS) is assigned to the rare diseases group caused by a complete or partial loss of the second X chromosome (45, X0) and lead with typical clinical features [1–3], with the incidence of 1 in 2500 live born females [4]. Morbidity and mortality of TS patients are high, particular due to cardiovascular disorders [2]. Associated congenital cardiovascular malformations are well-known in this population, with a prevalence ranging from 17 to 45% [2, 3]. The bicuspid aortic valve and coarctation of the aorta are the most common [1, 5]. Additionally, progressive dilation of the ascending aorta is observed in 20–39% of TS patients [6]. Even if the prevalence appears to increase with age, abnormal aortic dimensions are present since childhood [7]. Accurate assessment of the aorta is crucial in patients with TS because aortic dissections and ruptures occur more frequently than in the general population [8]. Edification of management in cardiovascular monitoring is necessary to avoid the diagnostic delay [9, 10]. It has been proven that magnetic resonance imaging (MRI) is a gold standard in the diagnosis of cardiovascular complications in TS patients and must be used in routine clinical practice for patients with TS [7, 11]. Still, 2-dimensional echocardiography (2Decho) remains a basic and reliable diagnostic test for a primary diagnosis of cardiovascular disorders. The data of different studies have demonstrated a strong correlation between 2Decho evaluation and MRI [11], but 2Decho is not a method of choice when evaluating the diameter of the overall aorta in TS patients; MRI must be performed instead. In our study, we aimed to compare aortic dimensions on MRI and 2Decho in TS patients.

## 2. Methods

In this cross-sectional study, we included all patients with genetically confirmed Turner syndrome followed up at the Department of Endocrinology, Hospital of Lithuanian University of Health Sciences, between 2014 and 2017. The sample consisted of 50 TS patients aged between 18 and 60 years. Patients who underwent focused echocardiograms and MRI were eligible. Patients younger then 18 years old and patients with missing important data were excluded from the study. The study protocol has been approved by the Kaunas Region Biomedical Research Ethics Committee (No. BE-2-32, 2014-06-03), according to the requirements of the Declaration of Helsinki.

Anthropometric measurements (height, weight) were assessed, and a basic clinical examination was performed. Body height was measured with a wall-mounted stadiometer, and body weight of barefoot patients was measured with an electronic scale. Body mass index (BMI) was calculated using a BMI formula (weight (kg)/height^2^ (m)).

Arterial hypertension was defined as blood pressure > 140/90 mmHg or requirement for antihypertensive therapy as defined by the American Heart Association. Blood pressure was measured 3 times on the right arm. The cuff was localized at the heart level.

### 2.1. 2D Echocardiography and Magnetic Resonance Imaging

Ascending aorta dimensions were assessed using two-dimensional echocardiography and thoracic MRI at the Hospital of Lithuanian University of Health Sciences.

Two-dimensional echocardiography was performed using a GE Vivid 7 system (GE Vingmed Ultrasound AS N-3190, Horten, Norway). Echocardiographic studies were performed by an independent experienced echocardiographer, blinded to the patient's clinical data. Digital loops were stored and analyzed offline (EchoPac V.6.0.0; GE Vingmed). Anatomic examinations and measurements were performed according to the American Society of Echocardiography recommendations. Maximal diameter of the sinuses of Valsalva (M1), diameter of the sinotubular junction (M2), and the proximal ascending aorta (M3) were measured at an end diastole from leading edge to leading edge in a perpendicular plane along the axis of the aorta. The aortic arch proximal to the left subclavian artery (M4) and the proximal part of the descending aorta (M5) were measured [12].

**Figure 1:**
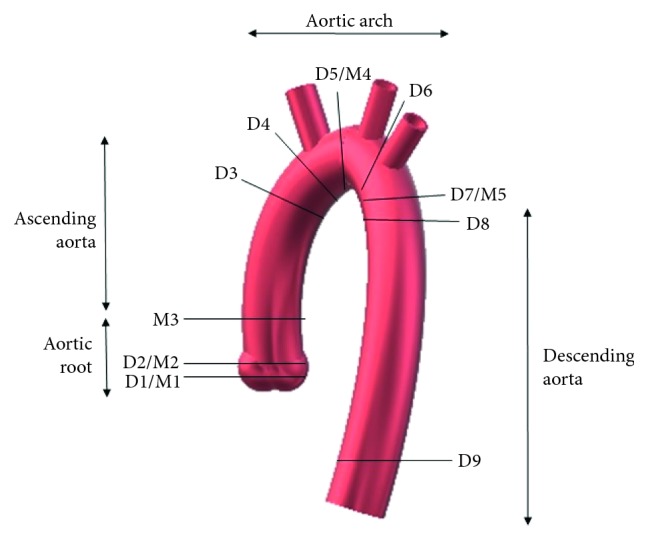
Corresponding points of the aorta in different levels measured by 2Decho and MRI.

Triggered breath-hold contrast-enhanced magnetic resonance angiography was performed on a 1.5 T MRI unit (Siemens Aera, Siemens AG, Erlangen, Germany), intravenously applying gadolinium-based contrast agent (Gadovist 1.0 mmol/ml, Bayer AG, Germany) with a dose of 0.1–0.15 mmol/kg, at a rate of 3.5 ml/s. The size of the ascending aorta was measured in nine different positions [13]: in the aortic sinus (D1), in the sinotubular junction (D2), in the ascending aorta at the bottom edge of the right pulmonary artery (D3), in the ascending aorta at the right proximal brachiocephalic artery (D4), in the proximal transverse aortic arch (D5), in the distal transverse aortic arch (D6), in the descending aorta in the isthmus region (D7), in the descending aorta at the level of the left pulmonary artery (D8), and the thoracoabdominal aorta at the level of the diaphragm (D9) (Figure 1). Perpendicular aortic diameters were measured on longitudinal axes in the end diastole.

The diameter of the ascending aorta was adjusted according to the body surface area since the reduced final height and reduced body surface area is similar in patients with TS [6, 14]; the aortic size index (ASI) was calculated. Body surface area was calculated according to the formula of Du Bois and Du Bois: body surface area (m^2^) = 0.20247 × height (m) 0.725 × weight (kg) 0.425. In patients with TS, aortic dilatation is defined as ASI ≥ 20 mm/m^2^ [6].

The absolute diameter of the arch of the aorta or descending aorta was included into the analysis [6]. The aortic dilation was described as the diameter of arch of the aorta or the diameter of the descending aorta 50% higher when compared with normal ranges of the aorta adjusted for age [15].

### 2.2. Statistical Analysis

The data were analyzed using SPSS v.25 statistical package. All continuous variables were indicated as means ± standard deviations. Frequencies and percentages represented the descriptive statistics for categorical variables, and mean ± standard deviation values were used for continuous variables. Ratios were compared using a chi-square test, and mean values were compared using a *t*-test; Student's and Pearson coefficients were calculated to identify the correlations between variables. A *p* value of < 0.05 was considered statistically significant, and *r* values were employed to describe the coefficient of correlation.

## 3. Results

50 TS (45, X—66%) patients, ≥18 years (mean age 29.7 ± 8.2, range 18–60 years), were enrolled into the study. Basic characteristics of the participants are overviewed in Table 1.

The prevalence of bicuspid aortic valve was 16%; the coarctation of the aorta was diagnosed in 4% of the patients. Arterial hypertension has been detected in 32% of patients.

ASI on echocardiography strongly correlated with ASI on MRI in all positions (Table 2) of the ascending aorta; still, the medians of the ASI were significantly different when comparing the two methods of assessment. Significantly larger medians of ASI were found on 2Decho in all positions of the ascending aorta and arch when compared with MRI measurements (Table 3).

Still, the prevalence of aortic sinus dilation was significantly and more frequently (52% vs. 38%, *p* < 0.001) observed on MRI when compared with 2Decho. Higher prevalence of aortic dilation in other positions was found on 2Decho compared with MRI (Table 4).

Age, congenital cardiovascular disorders, presence of hypertension, anthropometric measurements, and SHRT or previous treatment with GH did not interfere with the difference of the presence of the dilation of the aorta on MRI when compared with 2Decho.

## 4. Discussion

Our results demonstrate that the diameter of the aorta in MRI strongly correlates with 2Decho in all positions; still, MRI identifies dilatation of the sinus of aorta that was missed by 2Decho.

Monitoring for cardiovascular abnormalities is recommended for all patients with TS because of the potentially lethal complications [11, 16], but a proper cardiovascular follow-up is challenging in this population [17, 18]. Different recommendations for a proper cardiovascular follow-up in TS population still exist. A group of researchers have proven that MRI must be used in routine clinical practice for patients with TS [17, 19]; another study shows that 2Decho is a reliable diagnostic method [19]. Despite the significant strong correlation of aortic dimensions in all positions on MRI and 2 Decho, a higher prevalence of the dilatation of the sinus of aorta was observed on MRI compared with 2Decho. The recent guidelines [6] recommend to use MRI as a gold standard when evaluating diameter of the ascending aorta in specific TS population.

Generally, 2Decho has become the most common imaging test in the field of diagnostics of cardiovascular diseases. This method can be appropriate when performed by an expert cardiologist, but 2Decho is not the technique of choice for overall evaluation of the aorta. Still, it is proven that MRI allows a better visualization of the aorta and should be performed prior to 2Decho in the specific population of TS [4, 17, 20]. The value of 2Decho in the TS population is potentially suboptimal because of a difficult rendering of appropriate echocardiography acoustic windows due to an abnormal chest anatomy [6, 21].

Dilatation of the descending aorta is not well shown in echocardiography, and the frequency of those patients considered to have aortic dilatation is increased using MRI [16]. However, we did not find any dilatation of the descending aorta in the studied cohort. The study by Lanzarini et al. made a comparison of echocardiography and MRI measurements for aortic dimensions in TS patients of a different age. Statistically insignificantly different results were obtained by measuring an aortic root and the ascending thoracic aorta. Measurements in the rest of the aorta differed widely [22]. In the MRI study by Dawson-Falk et al. of 40 TS patients, the utility of echo and MRI were compared in the evaluation of only the ascending aorta. Five cases of dilatation were identified in this investigation, four of which would not have met criteria for dilatation based on echo alone [23]. A study in the USA has also shown that evaluation done using 2Decho is inefficient when detecting the aortic pathology; in this study, 2Decho evaluation has missed 17% of dilatations compared with MRI [21].

Some data suggest that the elongation of the aorta should be known as a risk factor for aortic dilation in TS [24]. This abnormality is an arterial finding in women with TS only detectable by MRI [17]. MRI reveals the prevalence of risk factors for dissection as BAV, coarctation, and elongation of the aorta. MRI often shows these defects after 2Decho evaluation has shown diameter and anatomy of the aorta to be normal [25].

In a primary echo study by Sachdev et al. of TS patients of different age, a part of whom underwent MRI to determine aortic valve morphology, the diameter of the aorta was also assessed. The results showed 11% of the study population to have dilatation. Simple regression of aortic measurements showed correlations between 2Decho evaluation and MRI values [26]. On the contrary, 2Decho has been proven to have the ability to identify valvular abnormalities better than MRI [24].

Recommendations of another study in the USA summarize our results and analysis; it is important to perform both 2Decho and MRI to ensure identification of all cardiac lesions [11].

Many other risk factors such as karyotype, presence of BAV, aortic coarctation, and hypertension are described to allow the increase in dimensions of the aorta in TS patients [14, 21, 24, 27–29]. Statistically significant correlation between 45, X karyotype, BAV, and aortic dilatation was confirmed in a French study [10]. In another study, a direct influence of karyotype on the diameter of aorta associated with the indirect influence via BAV and aortic coarctation has not been found [29]. Hypertension leads to increased risk and progression of aortic dilation; the growth rate of the aorta can be accelerated by hypertension [30, 31]. Correlation between the mentioned risk factors in our study has not been demonstrated, presumably due to a small sample of TS patients in our research.

## 5. Conclusion

The relation of aortic size index is significant in all positions when comparing measurements on MRI and 2Decho; still, the dilatation of the sinus of aorta is more frequently found on MRI compared with echocardiography.

A small sample size is a common limitation in the assessment of rare diseases. It is possible that delamination of blood moving near the wall falsely decreased the measured ASI on MRI.

## Figures and Tables

**Table 1:** Basic clinical characteristics of TS patients.

	Minimum	Maximum	Mean	Standard deviation
Weight (kg)	32	81	57.14	11.68
Height (cm)	137	169	152.14	6.49
BMI (kg/m^2^)	15.24	36.03	24.57	4.84
Heart rate (beats/min)	60	100	83	9.7
Systolic blood pressure (mmHg)	90	160	119	14.8
Diastolic blood pressure (mmHg)	60	100	78	12
Age at GH initiation (yrs)	2	17	11	3.8
Duration of GH treatment (yrs)	1	14	4.0^*∗*^	—
Age at estrogen initiation (yrs)	11	19	14^*∗*^	—

^*∗*^Median.

**Table 2:** The relation between the measurements of the aorta on 2Decho and MRI.

Position of the measurement	*r* value	*p* value
M1/D1	0.842	<0.001
M2/D2	0.819	<0.001
M3/D3	0.597	<0.001
M3/D4	0.620	<0.001
M4/D5	0.264	0.067
M4/D6	0.397	0.005
M5/D7	0.199	0.207
M5/D8	0.351	0.022
M5/D9	0.337	0.018

*r*: Spearman's correlation.

**Table 3:** Differences in medians of aortic dimensions measured by 2Decho and MRI.

	Median of ASI/diameter of aorta on 2Decho	Median of ASI/diameter of aorta on MRI	*p* value
M1/D1	20.04 (14–30) mm/m^2^	20.04 (12–30.5) mm/m^2^	0.795
M2/D2	18.36 (12.65–24.67) mm/m^2^	15.85 (10.15–22.59) mm/m^2^	<0.001
M3/D3	18.85 (14–29.22) mm/m^2^	17.3 (10.3–28) mm/m^2^	0.001
M3/D4		16.23 (10.75–26.24) mm/m^2^	<0.001
M4/D5	23 (16–40) mm	24.3 (14.9–45.7) mm	0.077
M4/D6		20.15 (14.5–28) mm	<0.001
M5/D7	17.5 (14–31) mm	18.1 (12–25.1) mm	0.591
M5/D8		17.8 (13–26.3) mm	0.574
M5/D9		16.4 (12.1–21.5) mm	0.003

The significance was calculated on the basis of the nonparametric Wilcoxon test for two related samples; min and max values are listed.

**Table 4:** Frequencies of aortic dilatation in different levels measured by 2Decho and MRI.

	Frequency of 2D echo indicated aortic dilatation (%)	Frequency of MRI indicated aortic dilatation (%)	*p* value
Sinus (M1/D1)	38	52	<0.001
Sinotubular junction (M2/D2)	18	10	0.004
Ascending aorta (M3/D3) (M3/D4)	32	22 12	0.001
			0.034
Aortic arch (M4/D5) (M4/D6)	2	4	0.835
		0	—
Descending aorta (M5/D7) (M5/D8) (M5/D9)	0	0	—
		0	
		0	

*p* value was calculated using the *χ*^2^ criterion.

## Data Availability

The data used to support the findings of this study are included within the article.
